# Cytogenetic markers as a tool for characterization of hybrids of *Astyanax* Baird & Girard, 1854 and *Hyphessobrycon* Eigenmann, 1907

**DOI:** 10.3897/CompCytogen.v14i2.49513

**Published:** 2020-05-27

**Authors:** Caio Augusto Gomes Goes, Sandro Natal Daniel, Lucas Henrique Piva, George Shigueki Yasui, Roberto Ferreira Artoni, Diogo Teruo Hashimoto, Fausto Foresti, Fábio Porto-Foresti

**Affiliations:** 1 Universidade Estadual Paulista (UNESP) “Júlio de Mesquita Filho”, Faculdade de Ciências, Edmundo Carrijo Coube Avenue, Bauru, SP, Brazil Universidade Estadual Paulista Bauru Brazil; 2 Centro nacional de Pesquisa e Conservação da Biota Aquática Continental (CEPTA-ICMBIO), Prefeito Euberto Nemésio Pereira Godói Highway, Pirassununga, SP, Brazil Centro nacional de Pesquisa e Conservação da Biota Aquática Continental Pirassununga Brazil; 3 Universidade Estadual de Ponta Grossa, Setor de Ciências Biológicas e da Saúde, Santos Andrade Square, Ponta Grossa, PR, Brazil Universidade Estadual de Ponta Grossa Ponta Grossa Brazil; 4 Universidade Estadual Paulista (UNESP) “Júlio de Mesquita Filho”, Centro de Aquicultura da UNESP, Prof. Paulo Donato Castelane Acess way, Jaboticabal, SP, Brazil Universidade Estadual Paulista Jaboticabal Brazil; 5 Universidade Estadual Paulista (UNESP) “Júlio de Mesquita Filho”, Instituto de Biociências, Prof. Montenegro Avenue, Botucatu, SP, Brazil Universidade Estadual Paulista Botucatu Brazil

**Keywords:** neotropical fishes, B chromosomes, chromosome polymorphism, repetitive DNAs, species complex

## Abstract

*Astyanax* Baird et Girard, 1854, is one of the largest genera in the family Characidae and comprises 177 valid species. This genus has been the focus of cytogenetic studies primarily owing to the presence of B chromosomes and high karyotypic diversity among different populations. The intense genetic variability in *Astyanax* is one of the factors responsible for the occurrence of species complexes, which are groups (1) with certain difficulties in establishing common genetic pools or (2) belonging to different cryptic species. To evaluate cytogenetic marker inheritance and the possibility of the identification of these hybrids, this study aimed to describe cytogenetic hybrids from three strains of species of the genera *Astyanax* and *Hyphessobrycon* Eigenmann, 1908. *A. lacustris* Lütken, 1875, *A. schubarti* Britski, 1964, *A. fasciatus* Cuvier, 1819, and *H. anisitsi* Eigenmann, 1907 were used to generate three hybrid lineages. The diploid number, heterochromatin sites, and ribosomal genes (18S and 5S rDNA) of the parental strains and the hybrids were analyzed. The results indicated that the three hybrid lineages had cytogenetic markers of both parents, presenting Mendelian inheritance. However, differences in distribution of heterochromatic blocks were observed between the hybrids and the parent strains. Our results allowed the identification of the hybrid strains based on the cytogenetic markers applied, reinforcing the efficiency of cytogenetic markers as tools for identification and indicating that such events may increase the karyotypic diversity in the genera *Astyanax* and *Hyphessobrycon*.

## Introduction

Interspecific hybridization is the union of distinct genetic pools, the progenies of which are usually individuals posessing intermediate taxonomic characteristics of both parental species (Mayr 1963). In fishes, hybridization is facilitated by reproductive peculiarities, such as external fertilization and sharing of spawning sites, which may eventually facilitate the occurrence of cross-fertilization and the emergence of hybrid strains (Hubbs 1955). Of note, sporadic cases of natural hybrids occur in Neotropical fish species (Artoni et al. 2006; Porto-Foresti et al. 2013; Hashimoto et al. 2014; Prado et al. 2017).

*Astyanax* Baird et Girard, 1854, belonging to the family Characidae, is one of the most species-rich genus and currently comprises 177 valid species (Eschmeyer and Fong 2020), known as tetras. The genus *Astyanax* is characterized by high phenotypic plasticity and a capacity to adapt to diverse habitats (Ornelas-Garcia et al. 2008). Cytogenetic data available for this genus reveal wide karyotypic diversity with exclusive chromosomal features of some species and populations, such as the presence of heterochromatin polymorphisms and distinct patterns of repetitive DNA dispersion (Mantovani et al. 2000; Almeida-Toledo et al. 2002; Kantek et al. 2009; Hashimoto and Porto-foresti 2010; Hashimoto et al. 2011; Utsunomia et al. 2017). These intense genetic polymorphisms result in several “species complexes,” described as a cluster of closely related populations, the individuals of which may represent more than one species (Fegan and Prior 2005). In the genus *Astyanax*, species complexes have been described in at least four species: *A. scabripinnis* Jenyns, 1842 (Moreira-Filho 1991), *A. lacustris* Lütken, 1875 (Fernandes and Martins-Santos 2004), *A. fasciatus* Cuvier, 1819 (Artoni et al. 2006), and *A. bimaculatus* Linnaeus, 1758 (Garutti and Langeani 2009). In these cases, different natural isolated populations of individuals with similar morphology considered as a unique species may not share the same cytogenetic markers or diploid number. In these cases, it is very difficult to define whether they share the same gene pool or if they are different cryptic species. In addition to the intense chromosomal polymorphisms, the possibility of the occurrence of hybrids in the natural environment can increase karyotypic diversity and complicate the accurate identification of the animals.

There has been a report of interspecific hybridization among *Astyanax* species in the nature (Pazza et al. 2006). Thus, the occurrence of natural hybrids in *Astyanax* populations is a factor to be considered in the cytogenetic studies concerning this genus. Considering the importance of using efficient tools in the identification of hybrids, the objective of this study was to, for the first time, cytogenetically describe the hybrids of two strains between species of *Astyanax* and a strain between a species of *Astyanax* and a species of the genus *Hyphessobrycon* to observe the inheritance of cytogenetic markers from the parent stains. The study also aimed to verify the possibility of identifying a hybrid using cytogenetic markers, to contribute to the understanding of the evolutionary dynamics of the group.

## Material and methods

The parent strains used in this study were obtained from the Instituto Chico Mendes de Conservação da Biodiversidade (CEPTA – ICMBIO/Pirassununga, SP, Brazil), where artificial crossing was performed. The crosses were directed using *A. lacustris* females and *A. fasciatus*, *A. schubarti* Britski, 1964, and *H. anisitsi* Eigenmann, 1907, males. Ovulation was induced in *A. lacustris* using the protocol established by Yasui et al. (2015), and spermatogenesis in males of the other species was induced with a single dose of carp pituitary gland (5 mg kg^-1^). The gametes were collected by stripping, the oocytes were stripped on a plastic Petri dish and the sperm was collected using a 1000 µl micropipette and transferred to a tube containing 300 µl of Ringer solution (Piva et al. 2018). Oocytes fertilization was initiated in the Petri dish using 80µl of sperm from selected males, and gamete activation was achieved by adding 5ml of water followed by immediate mixing via gentle hand movements.

The hybrids were identified and deposited in the Laboratório de Genética de Peixes, Bauru, São Paulo, Brazil, under the accession numbers LGP8291–LGP8382. Fifty-nine animals were anesthetized using 1% benzocaine. Mitosis stimulation was performed using the method described by Oliveira et al. (1988). Subsequently, mitotic chromosomes were obtained from kidney tissue using protocols described by Foresti et al. (1981) and Foresti et al. (1993). Seventeen hybrids of *A. lacustris* × *A. fasciatus*, 10 of *A. lacustris* × *A. schubarti*, and 32 of *A. lacustris* × *H. anisitsi* were analyzed. C-positive heterochromatin was detected using the barium hydroxide method (Sumner 1972). Chromosomes were classified as metacentric (m), submetacentric (sm), subtelocentric (st), and acrocentric (a) according to their arm ratios (Levan et al. 1964).

5S (two different bands: 255 and 525 bp) and 18S (one band: 600 pb) rDNA probes were obtained using polymerase chain reaction with the primers 5S A (5'-TCAACCAACCACAAAGACATTGGCAC-3') and 5S B (5'-TAGACTTCTGGGTGGCCAAAGGAATCA-3') for the 5S gene (Pendás et al. 1994) and 18S A (5'-TACGCCCGATCTCGTCCGATC-3') and 18S B (5'-CAGGCTGGTATGGCCGTAAGC-3') for the 18S gene (Utsunomia et al. 2016). For fluorescence in situ hybridization, chromosomes were treated following the protocol described by Pinkel et al. (1986). The probes were labeled using biotin-14-dATP and digoxigenin-11dUTP (Roche Applied Science) and the signals were detected using avidin-fluorescein conjugate (FITC) and anti-digoxigenin-rhodamine, respectively. Images were captured using Olympus QColor coupled to a fluorescence photomicroscope (BX50, Olympus), and the images were processed using the CellSens Standard Software.

## Results

**Figure 1.**
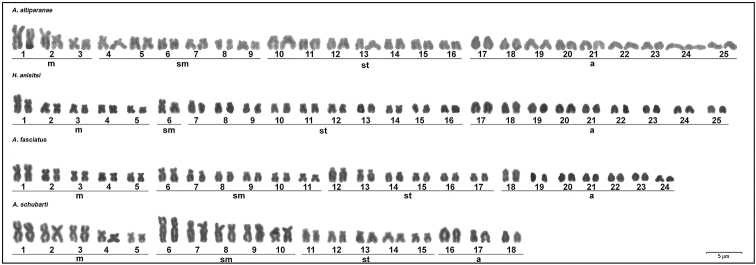
Karyotypes of the parental individuals analyzed: *Astyanax lacustris* (3m+6sm+7st+9a), *Hyphessobrycon anisitsi* (5m+1sm+10st+9a), *A. fasciatus* (5m+6sm+6st+7a), and *A. schubarti* (5m+5sm+5st+3a). Scale bar: 5 µm.

**Figure 2.**
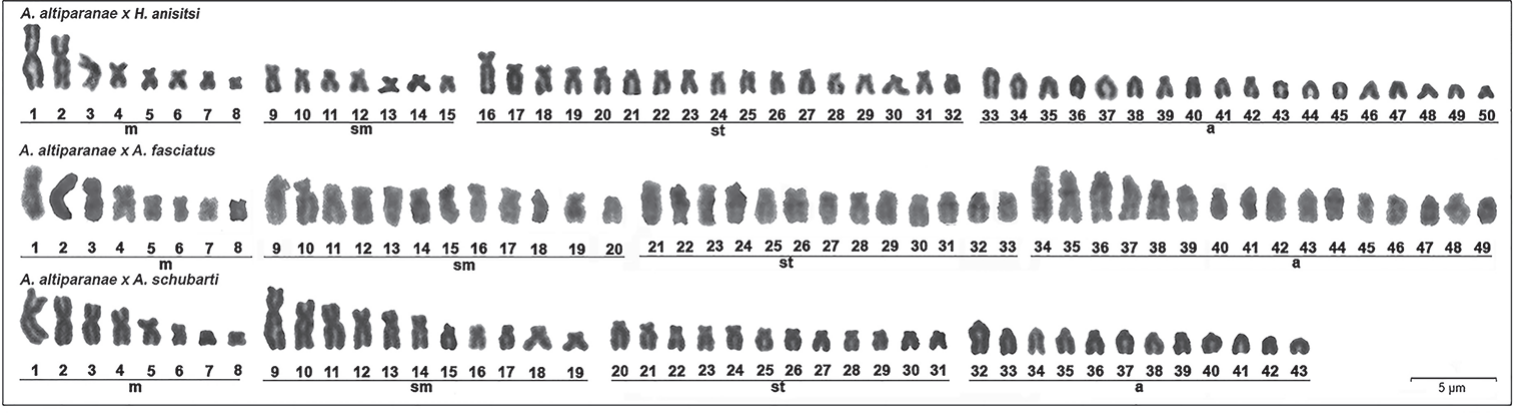
Karyotypes of three hybrids of species of the genus *Astyanax*: *A. lacustris* × *Hyphessobrycon anisitsi* (8m+7sm+17st+18a), *A. lacustris* × *A. fasciatus* (8m+7sm+17st+18a), and *A. lacustris* × *A. schubarti* (8m+11sm+12st+12a). Scale bar: 5 µm.

**Figure 3.**
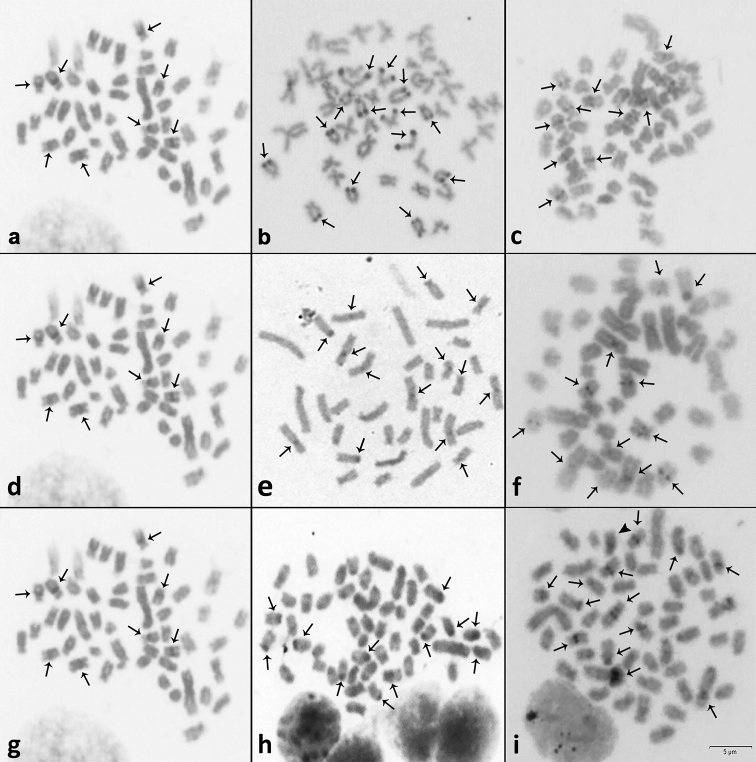
Heterochromatic markers obtained by C-banding on metaphase plates of *Astyanax lacustris* (**a**), *A. fasciatus* (**b**), and *A. schubarti* (**c**), and *Hyphessobrycon anisitsi* (**d**) and hybrids *A. lacustris* × *A. fasciatus* (**e**), *A. lacustris* × *A. schubarti* (**f**), and *A. lacustris* × *H. anisitsi* (**g, h**) after C-banding. The arrows indicate heterochromatic markers. In **h**, a metaphase with 51 chromosomes, the chromosome being completely heterochromatic, can be observed. Scale bar: 5µm.

All parent strains displayed stable diploid chromosome numbers; *A. lacustris* displayed 2n = 50 (6m+12sm+14st+18a) chromosomes; *A. fasciatus*, 2n = 48 (10m+12sm+12st+14a); *A. schubarti*, 2n = 36 (10m+10sm+10st+6a); and *H. anisitsi*, 2n = 50 (10m+2sm+20st+18a) (Fig. 1). The *A. lacustris* × *A. fasciatus* progeny displayed 49 chromosomes (8m+12sm+13st+16a) and the *A. lacustris* × *A. schubarti* progeny displayed 43 chromosomes (8m+11sm+12st+12a); the *A. lacustris* × *H. anisitsi* progeny displayed chromosome number variation, with some individuals showing 50 or 51 chromosomes (Fig. 2). Importantly, this extra chromosome (from individuals showing 51 chromosomes) was C-band positive, different from the regular set of chromosomes (Fig. 3).

The results of C-positive heterochromatin revealed some interesting features. *Astyanax lacustris* and *A. schubarti* hybrids showed regular heterochromatic blocks inherited from both parent strains. The terminal heterochromatic blocks in subtelocentric/acrocentric chromosomes of *A. fasciatus* and the typical location of As51 satellite DNA were not detected in the hybrids (Figure 3); furthermore, the *A. lacustris* × *H. anisitsi* hybrids displayed a conspicuous heterochromatic block in the *p* arm of the large subtelocentric chromosome, and this was not detected in any parent strain (Fig. 3).

**Figure 4.**
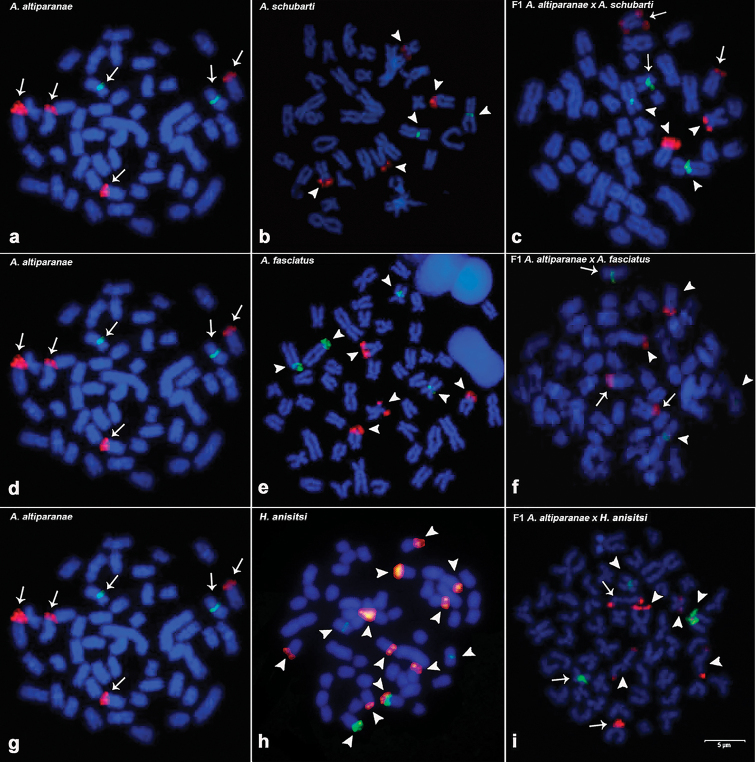
Fluorescence *in situ* hybridization with the probes DNAr 5S (green) and 18S (red). The results are labeled as: *Astyanax lacustris* (**a, d, g**), *A. schubarti* (**b**), hybrid *A. lacustris* × *A. schubarti* (**c**), *A. fasciatus* (**e**), hybrid *A. lacustris* × *A. fasciatus* (**f**), *Hyphessobrycon anisitsi* (**h**), and hybrid *A. lacustris* × *H. anisitsi* (**i**). Arrows and arrowheads indicate chromosomes bearing 18S and 5S rDNA clusters: arrows, *A. lacustris*; arrowheads, other species in the cross. Scale bar: 5µm.

**Figure 5.**
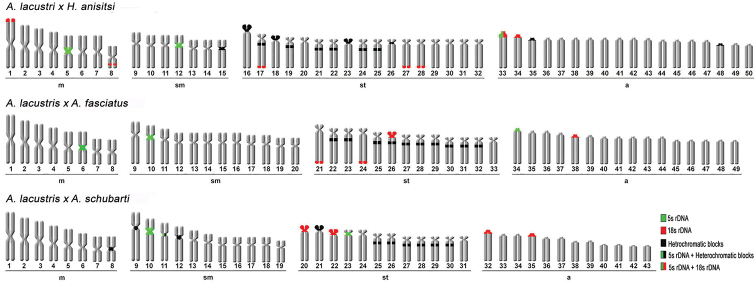
Ideogram of parental strains.

**Figure 6.**
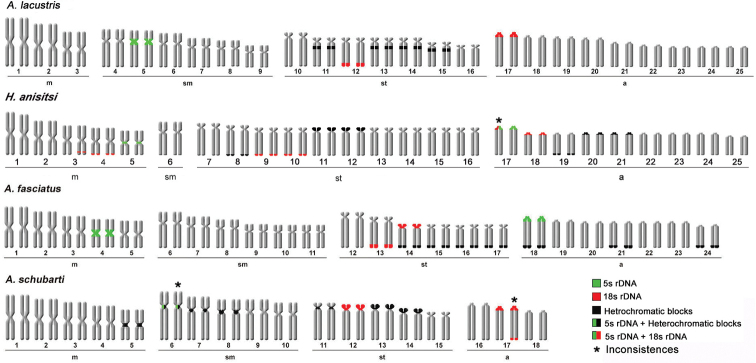
Ideogram of hybrid strains.

The ribosomal sites showed Mendelian inheritance, as revealed in Figure 4. Astyanax lacustris and A. schubarti displayed four sites of 18s rDNA and two sites of 5s rDNA. *Astyanax fasciatus* showed four sites of both markers, and *H. anisitsi* showed intense dispersion of 18s rDNA, with 10 sites of this marker. This species demonstrated four sites of 5s rDNA, one of them syntenic with 18s rDNA. In general, the hybrids demonstrated the inheritance of cytogenetic markers as expected, with some inconsistency in the *A. lacustris* × *A. schubarti* hybrid, as indicated by the observation of three sites of 5s rDNA instead of just two and a bi-telomeric site of 18S rDNA in an acrocentric chromosome. All cytogenetic analysis is resumed in ideograms of parent (Fig. 5) and hybrid (Fig. 6) strains.

## Discussion

The genus *Astyanax* is rich in chromosomal polymorphisms (Moreira-Filho 1991; Fernandes and Martins-Santos 2004; Artoni et al. 2006; Garutti and Langeani 2009). Moreover, the results of the present study are consistent with the literature regarding diploid numbers and the distribution of cytogenetic markers in the species used as parent strains (Mantovani et al. 2000; Almeida-Toledo et al. 2002; Kantek et al. 2009; Hashimoto and Porto-foresti 2010; Hashimoto et al. 2011). As expected, the resulting hybrids showed typical karyotypic features, inherited from the distinct parental strains.

Hybridization between different fish species can generate individuals that diverge from simple diploids with equal parental contribution (Toledo-Filho et al. 1994); andro or gynogenetic offspring as well as haploid, triploid, or tetraploid animals can be obtained. In the present study, it was possible to characterize all the strains as single diploid offspring because we identified the haploid sets from both parent strains involved in the crossing, resulting in diploid numbers intermediate to those of the parent strains.

The C-banding patterns revealed interesting features, as conspicuous heterochromatic blocks did not appear to be regularly inherited in some cases, indicating some degree of chromatin remodeling, similar to that in plant and mammal hybrids (O’neill et al. 1998; Comai et al. 2003). In both cases, heterochromatin expansion occurred through hypomethylation of genomic regions containing transposable elements, allowing for expansion of these mobile sequences. Considering the heterochromatic areas of tetras are mainly composed of transposable elements (Vicari et al. 2008; Silva et al. 2013; Barbosa et al. 2017), it can be hypothesized that hybridization affects these regions within a single generation. Some inconsistencies were detected in the analysis of rDNA: an additional 5S rDNA site and a bi-telomeric 18S rDNA site in the *A. lacustris* × *A. schubarti* hybrid, synteny of the 5S and 18S genes in only one *H. anisitsi* chromosome [also observed in the *A. lacustris* × *H. anisitsi* hybrid, likely due to an intraspecific polymorphism of 18S rDNA distribution in *H. anisitsi* (Fig. 4), and an extra and totally heterochromatic chromosome in two *A. lacustris* × *H. anisitsi* hybrids (present in approximately 50% of analyzed cells). A case of B chromosomes totally heterochromatic from interspecific hybridization has been reported in fishes (Schartl et al. 1995); however, more studies are necessary to verify the hypothesis of this aneuploidy being a B chromosome.

Fertile hybrids have been described for different Neotropical fish species such as hybrids of the catfishes “cachapinta” and “pintachara,” *Pseudoplatystoma corruscans* (Spix et Agassiz, 1829) and *P. reticulatum* Eigenmann et Eigenmann, 1889, (Hashimoto et al. 2013; Prado et al. 2017) and those involving the Characiformes species *Piaractus mesopotamicus* (Holmberg, 1887), *Colossoma macropomum* (Cuvier, 1816), and *Piaractus brachypomus* (Cuvier, 1818) (Hashimoto et al. 2014). The fertility of the hybrids is a problematic issue owing to the extensive production of hybrids in Brazilian aquaculture and the recurrent escapes of these individuals to the nature, which threatens the maintenance of natural populations that are susceptible to backcrossing; contamination of their gene pools is also possible. In a recent study, using the same brood stock analyzed herein, Piva et al. (2018) stated that a complete sterile offspring was restricted to *A. lacustris* × *A. fasciatus* crossing. Surprisingly, offspring from distinct genera (*A. lacustris* × *H. anisitsi*) and those displaying highly differentiated karyotypes (*A. lacustris* × *A. schubarti*) showed normal gametogenesis. However, the possibility of viable gamete formation by these individuals and consequently their effective fertility can be affected owing to the unstable diploid number in some of the hybrid strains observed in this study, such as the *A. lacustris* × *A. schubarti* (2n = 43) hybrid, unlike other fertile natural hybrids resulting from parent strains with the same diploid number, as observed in hybrids of the catfishes “pintachara” and “cachapinta” (Prado et al. 2012).

## Conclusion

The cytogenetic markers applied to the hybrid strains analyzed in this study were efficient in terms of identification based on the known karyotype of the parent strains, which differentiates the hybrids involving species of the genus *Astyanax* from other hybrids of Neotropical fish, which keeps its cytotypes conserved (Prado et al. 2012). In this sense, the diploid number was especially helpful in detecting hybrids. In cases wherein the hybrid had the same diploid number as the parent strains, 5s rDNA was the best marker. This study describes, for the first time, three hybrid strains involving species of the genera *Astyanax* and *Hyphessobrycon* and shows the efficiency of cytogenetic markers in their identification. The results presented herein will contribute to future cytogenetic and evolutionary studies involving these genera aimed at karyotypic diversity and species complex formation; the present study also highlights the possibility of the use of cytogenetic markers in the identification of hybrids.
