# Corrigenda: Cytogenetic markers as a tool for characterization of hybrids of *Astyanax* Baird & Girard, 1854 and *Hyphessobrycon* Eigenmann, 1907. Comparative Cytogenetics 14(2): 231–242. https://doi.org/10.3897/CompCytogen.v14i2.49513

**DOI:** 10.3897/CompCytogen.v14i4.56080

**Published:** 2020-12-29

**Authors:** Caio Augusto Gomes Goes, Sandro Natal Daniel, Lucas Henrique Piva, George Shigueki Yasui, Roberto Ferreira Artoni, Diogo Teruo Hashimoto, Fausto Foresti, Fábio Porto-Foresti

**Affiliations:** 1 Universidade Estadual Paulista (UNESP) “Júlio de Mesquita Filho”, Faculdade de Ciências, Edmundo Carrijo Coube Avenue, Bauru, SP, Brazil Universidade Estadual Paulista Bauru Brazil; 2 Centro nacional de Pesquisa e Conservação da Biota Aquática Continental (CEPTA-ICMBIO), Prefeito Euberto Nemésio Pereira Godói Highway, Pirassununga, SP, Brazil Centro nacional de Pesquisa e Conservação da Biota Aquática Continental Pirassununga Brazil; 3 Universidade Estadual de Ponta Grossa, Setor de Ciências Biológicas e da Saúde, Santos Andrade Square, Ponta Grossa, PR, Brazil Universidade Estadual de Ponta Grossa Ponta Grossa Brazil; 4 Universidade Estadual Paulista (UNESP) “Júlio de Mesquita Filho”, Centro de Aquicultura da UNESP, Prof. Paulo Donato Castelane Acess way, Jaboticabal, SP, Brazil Universidade Estadual Paulista Jaboticabal Brazil; 5 Universidade Estadual Paulista “Júlio de Mesquita Filho” (UNESP), Instituto de Biociências, Prof. Montenegro Avenue, Botucatu, SP, Brazil Universidade Estadual Paulista Botucatu Brazil

## Abstract

Astyanax Baird et Girard, 1854, is one of the largest genera in the family Characidae and comprises 177 valid species. This genus has been the focus of cytogenetic studies primarily owing to the presence of B chromosomes and high karyotypic diversity among different populations. The intense genetic variability in Astyanax is one of the factors responsible for the occurrence of species complexes, which are groups (1) with certain difficulties in establishing common genetic pools or (2) belonging to different cryptic species. To evaluate cytogenetic marker inheritance and the possibility of the identification of these hybrids, this study aimed to describe cytogenetic hybrids from three strains of species of the genera Astyanax and Hyphessobrycon Eigenmann, 1908. A. lacustris Lütken, 1875, A. schubarti Britski, 1964, A. fasciatus Cuvier, 1819, and H. anisitsi Eigenmann, 1907 were used to generate three hybrid lineages. The diploid number, heterochromatin sites, and ribosomal genes (18S and 5S rDNA) of the parental strains and the hybrids were analyzed. The results indicated that the three hybrid lineages had cytogenetic markers of both parents, presenting Mendelian inheritance. However, differences in distribution of heterochromatic blocks were observed between the hybrids and the parent strains. Our results allowed the identification of the hybrid strains based on the cytogenetic markers applied, reinforcing the efficiency of cytogenetic markers as tools for identification and indicating that such events may increase the karyotypic diversity in the genera Astyanax and Hyphessobrycon.

After the publication of our article, we detected some inconsistencies in figures and figure captions. The nominal species *Astyanax altiparanae* was recently recognized as a new junior synonym of *Astyanax lacustris* Lütken, 1875. Thus, we corrected this issue in all figures. Captions of figure 3, 5 and 6 were also incorrect. Corrected figures and captions are as follows:

**Figure 1.**
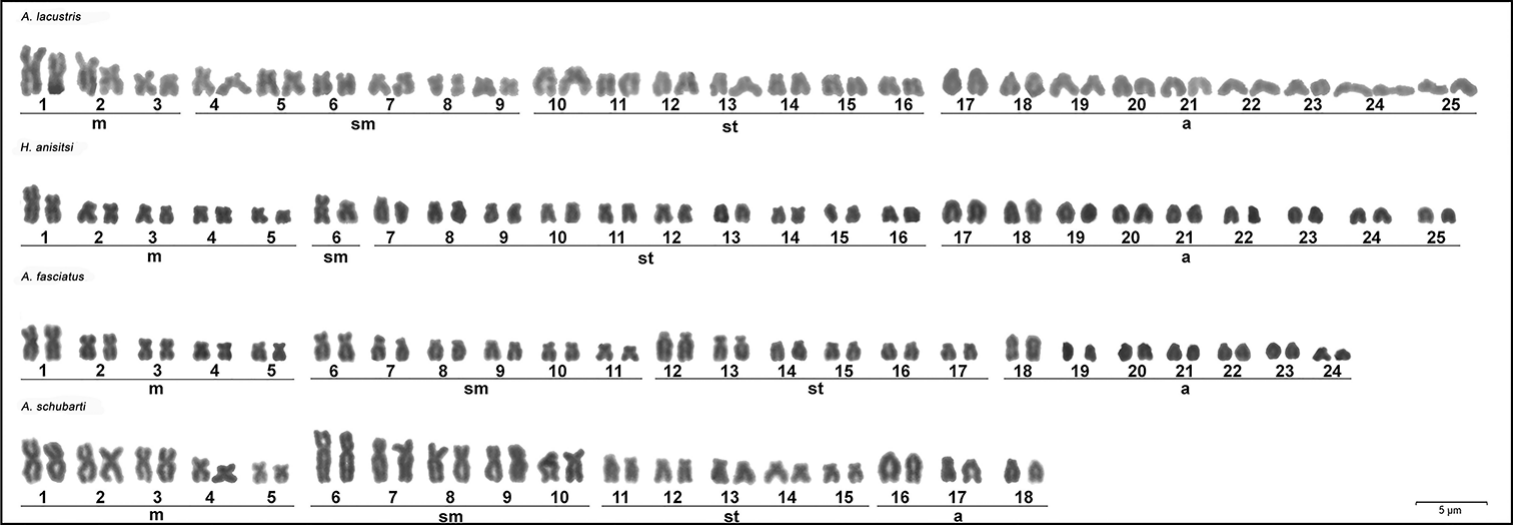
Karyotypes of parental individuals analyzed: *A. lacustris* (3m+6sm+7st+9a), *H. anisitsi* (5m+1sm+10st+9a), *A. fasciatus* (5m+6sm+6st+7a) and *A. schubarti* (5m+5sm+5st+3a). Scale bar: 5µm.

**Figure 2.**
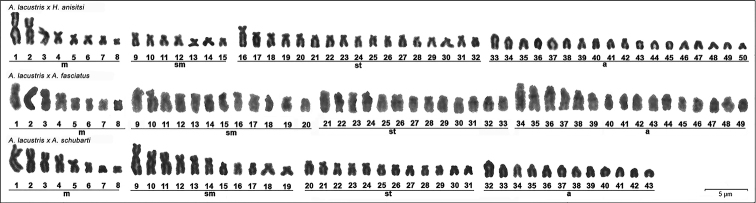
Karyotypes of hybrid products of the genus *Astyanax*: *A. lacustris* × *H. anisitsi* (8m+7sm+17st+18a), *A. lacustris* × *A. fasciatus* (8m+7sm+17st+18a) and *A. lacustris* × *A. schubarti* (8m+11sm+12st+12a), respectively. Scale bar: 5µm.

**Figure 3.**
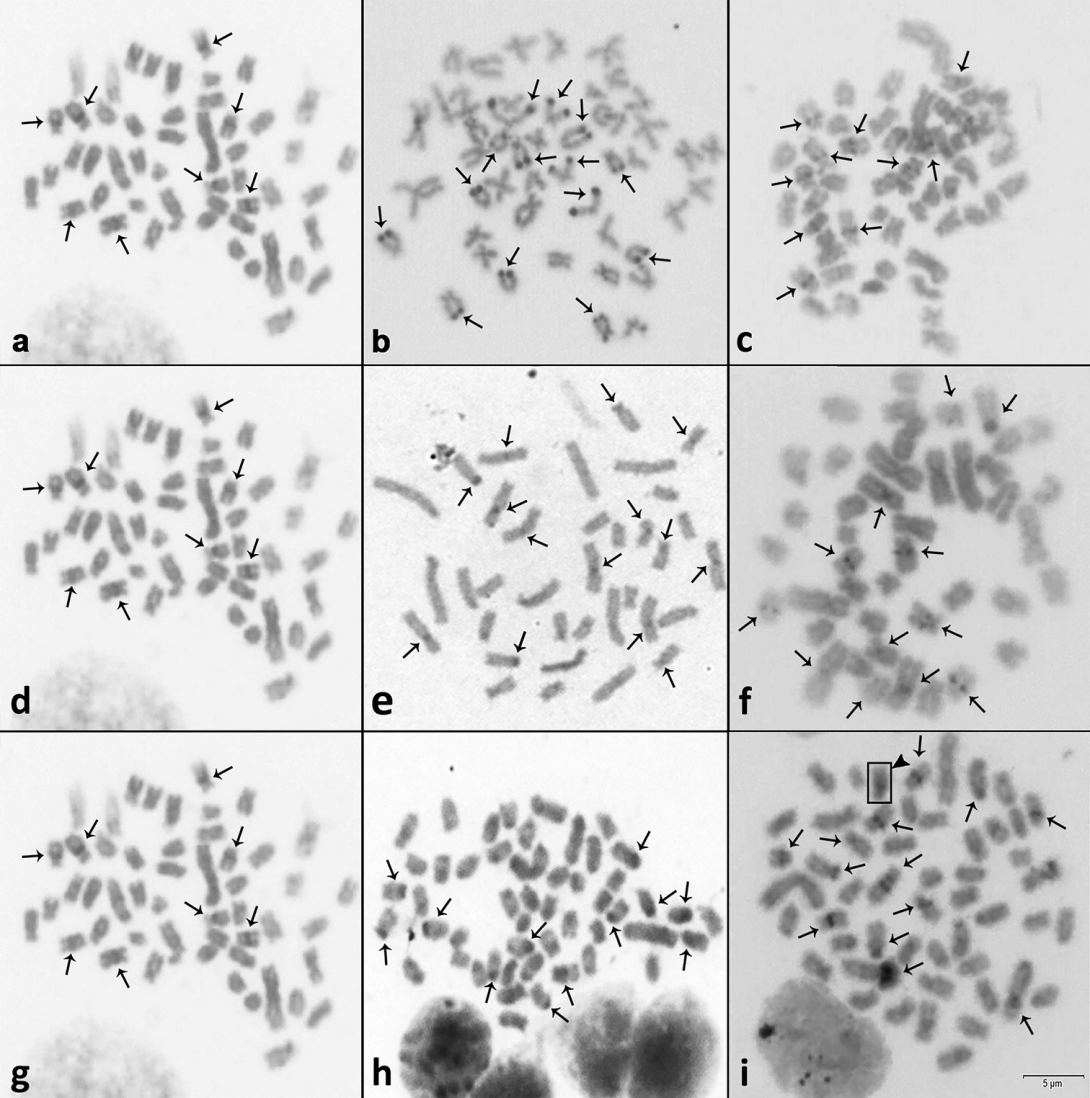
Heterochromatic markers obtained by C-banding on metaphase plates of *A. lacustris* (**a, d, g**), *A. fasciatus* (**b**), *A. schubarti* (**e**), *H. anisitsi* (**h**) and hybrids *A. lacustris* × *A. fasciatus* (**c**), *A. lacustris x A. schubarti* (**f**) and *A. lacustris* × *H. anisitsi* (**i**) after C-banding. The arrows indicate heterochromatic blocks. The chromosomes number of *A. lacustris* and *H. anisitsi* hybrid is 2N = 51, the metaphase plate of *A. lacustris* and *H. anisitsi* hybrid (i) contains a heterochromatic chromosome (arrowhead). Scale bar: 5µm.

**Figure 4.**
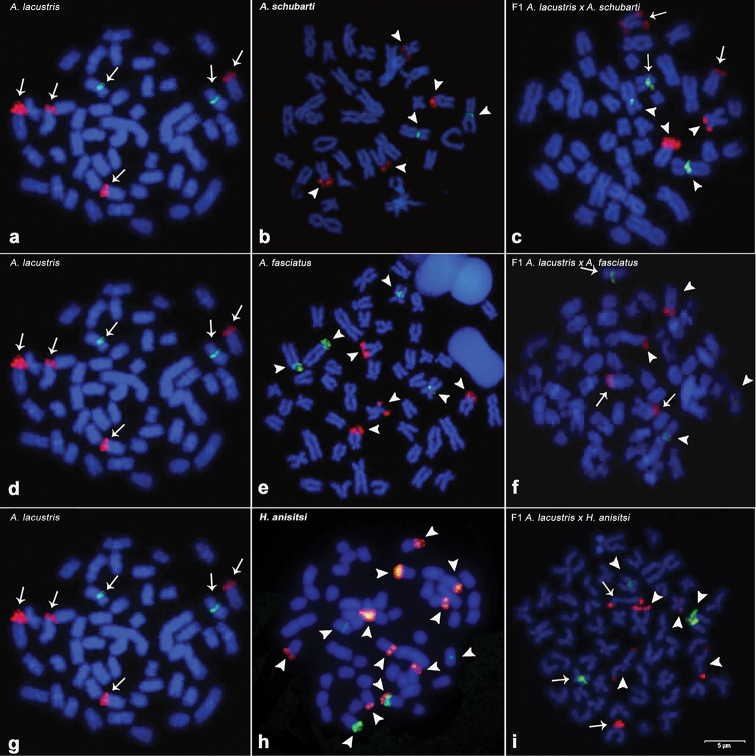
*FISH* with probes 5S (green) and 18S (red). The results are labeled as: *A. lacustris* (**a, d, g**), *A. schubarti* (**b**), hybrid *A. lacustris* × *A. schubarti* (**c**), *A. fasciatus* (**e**), hybrid *A. lacustris* × *A. fasciatus* (**f**), *H. anisitsi* (**h**), and hybrid *A. lacustris* × *H. anisitsi* (**i**). The arrows indicate chromosomes inherited from *A. lacustris*, and the arrowheads indicate chromosomes inherited from the other respective parents. Scale bar: 5µm.

**Figure 5.**
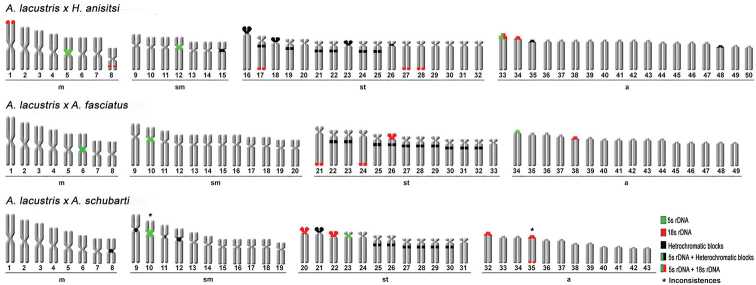
Ideogram of hybrids strains.

**Figure 6.**
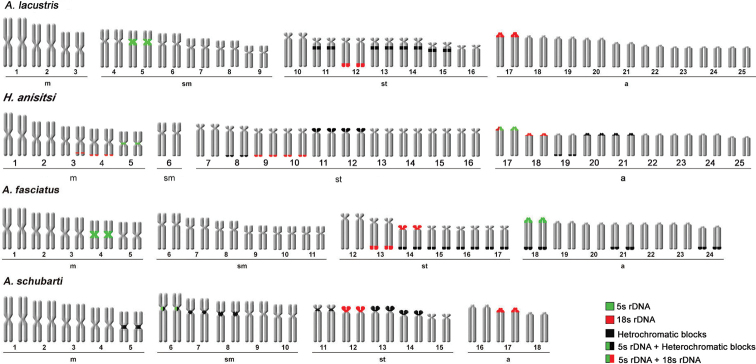
Ideogram of parental strains.

